# Single-Dose Microparticle Delivery of a Malaria Transmission-Blocking Vaccine Elicits a Long-Lasting Functional Antibody Response

**DOI:** 10.2174/1566524011313040002

**Published:** 2013-05

**Authors:** R.R Dinglasan, J.S Armistead, J.F Nyland, X Jiang, H.Q Mao

**Affiliations:** 1 W. Harry Feinstone Department of Molecular Microbiology & Immunology, Johns Hopkins Bloomberg School of Public Health, 615 N. Wolfe Street, Baltimore, MD 21205, USA; 2 Department of Pathology, Microbiology & Immunology, University of South Carolina School of Medicine, 6439 Garner's Ferry Road, Columbia, SC 29209, USA; 3 Department of Materials Science and Engineering, Johns Hopkins University, 3400 North Charles Street, Baltimore, MD 21218, USA; 4 Translational Tissue Engineering Center, Whitaker Biomedical Engineering Institute, Johns Hopkins School of Medicine, 400 North Broadway, Baltimore, MD 21287, USA

**Keywords:** Antigen, controlled release, immunity, malaria, midgut, mosquito, nanotechnology, natural boosting, sexual stages, transmission-blocking vaccine.

## Abstract

Malaria sexual stage and mosquito transmission-blocking vaccines (SSM-TBV) have recently gained prominence as a necessary tool for malaria eradication. SSM-TBVs are unique in that, with the exception of parasite gametocyte antigens, they primarily target parasite or mosquito midgut surface antigens expressed only inside the mosquito. As such, the primary perceived limitation of SSM-TBVs is that the absence of natural boosting following immunization will limit its efficacy, since the antigens are never presented to the human immune system. An ideal, safe SSM-TBV formulation must overcome this limitation. We provide a focused evaluation of relevant nano-/microparticle technologies that can be applied toward the development of leading SSM-TBV candidates, and data from a proof-of-concept study demonstrating that a single inoculation and controlled release of antigen in mice, can elicit long-lasting protective antibody titers. We conclude by identifying the remaining critical gaps in knowledge and opportunities for moving SSM-TBVs to the field.

## INTRODUCTION

The malaria eradication research agenda has re-emphasized the need for effective sexual stage and mosquito transmission-blocking vaccines (SSM-TBV) [1], which prevents malaria parasite development in its mosquito vector and the subsequent cascade of secondary infections [2-5]. SSM-TBVs, in general, work through the action of inhibitory antibodies [5-7]. Thus, the minimum objective of immunization is to induce high titer antibodies sustainable for at least one transmission season (~3-6 months), but preferably for 2 years. Achieving this minimum goal would theoretically drive the case reproductive rate, (R_0_) <1. A summary of the target product profile (TPP) for SSM-TBVs is shown in Table **1**. With the exception of *Plasmodium falciparum *or *P. vivax *gametocyte surface antigens that are expressed in the human, SSM-TBVs are considered unique in that they target parasite (gamete, zygote, or ookinete) or *Anopheles* mosquito midgut surface antigens that are only expressed in the mosquito. As such, one of the potential limitations of the TBV approach is that since the antigens are never naturally presented to the human immune system, the absence of natural boosting following immunization will limit their efficacy [8-13]. A complete *P. knowlesi *model in non-human primates (NHP) has been used to test the “natural boosting” hypothesis for *Plasmodium *gamete antigens [13]. It was found that following a two-dose immunization regimen using 10^5^-10^7^ *P. knowlesi *microgametes and macrogametes in a Freund’s complete adjuvant (FCA), the majority of the monkeys maintained a high level of functional transmission-blocking antibody titer for more than 1 year. Furthermore, annual challenge infections over a six year period were found to be sufficient for boosting and transmission-blocking immunity persisted in the majority of splenectomized NHPs. Importantly, as expected, they observed that transmission-blocking activity waned over time in the absence of boosting and that the challenge infection resulted in an increase in gamete-specific antibody levels. Although the likely gamete antigens had not yet been fully characterized at the time of this study, it was already known that gametocytes and gametes shared surface antigens [14, 15], thus it is possible that gametocyte exposure in the NHPs following challenge was responsible for boosting. This study further supported the notion that boosting would increase the efficacy and utility of SSM-TBVs but raised the question of the need for highly potent adjuvants such as FCA, which is considered a serious obstacle in human vaccine development.

The four leading SSM-TBVs (Table **2**) include two gametocyte surface antigens, Pfs230 [16-20] and Pfs48/45 [21], the ookinete surface protein Pfs25 [22] and the *Anopheles gambiae* alanyl aminopeptidase N (APN1), which is an abundant, midgut-specific apical microvilli surface glycoprotein that has been shown to mediate ookinete invasion and oocyst development [7, 23]. Of these, only Pfs25 and APN1 are expressed explicitly inside the mosquito midgut. Note that the goal of this report is not to evaluate the complete repertoire of proven and possible SSM-TBV candidates, and the reader is directed to several excellent reviews for additional information [3, 4, 24-29]. Among the four leading candidates, only Pfs25 has completed Phase I clinical trials, albeit with equivocal results [29]. Efforts are underway to produce the full-length Pfs/Pvs230 [30-32] and Pfs48/45 antigens [33-35], which have proven to be a difficult undertaking using different expression platforms due to their size and/or conformation, as well as the high A+T content of plasmodial genes; and these issues have a direct impact on vaccine process development. The APN1 antigen, on the other hand, does not require the full-length antigen, is highly immunogenic [7] and is entering process development, with an optimistic initiation of Phase I clinical trials within the next 3-4 years. Since Pfs25 and APN1-based vaccines are the least likely to benefit from boosting following natural infection, we focused on these two antigens in this article to examine their current state of development, as well as similarities and differences in the context of several identified target product profiles and the “natural boosting” issue (Table **1**). Furthermore, we have also used APN1 as a model antigen to directly address the above issue using nano- and microparticle technologies.

An ideal SSM-TBV formulation with a highly immunogenic antigen must therefore have the following characteristics: (i) it should be safe; (ii) it should not require a cold-chain; (iii) it should easily be administered; and (iv) a single immunization should confer long-lasting protection. A biodegradable microparticle (BMP) system, which provides sustained release of antigen and adjuvant properties, is capable of meeting these challenges. Several recent studies have demonstrated the utility of this general vaccine approach in vertebrate models [36-40]. Microparticle size is an important determinant for cell uptake [41, 42] and may also influence the antigen release rate [43]. In line with this, recent studies have shown that smaller particle delivery systems are effective in eliciting a robust immune response to the target immunogen [44-47]. The bioabsorption rate of BMPs and antigen release rate can be engineered to provide boosting from weeks to several months. Particles carrying single or multiple antigens can arguably mimic viral antigen presentation thus rapidly inducing a potent and long-lasting cellular and humoral response either by direct immune stimulation of antigen presenting cells (APCs) or/and by delivering antigen to the lymph node [30, 37, 48]. In fact, virosomes follow this approach and have shown to be effective carriers for proteins and subunit vaccines against a variety of pathogens, including malaria [49], but to date, this approach has not been used to deliver SSM-TBV antigens. With these goals in mind, we conducted proof-of-concept studies to test the hypothesis that safe biodegradable microparticles can mimic natural boosting through sustained release of antigen and, in doing so, elicit significant transmission-blocking antibodies against *Plasmodium*.

## MATERIALS AND METHODS

### Preparation of Biodegradable Microparticles (BMPs) with Different Size Range and Different Antigen Loading Levels

**Fig. (1):**
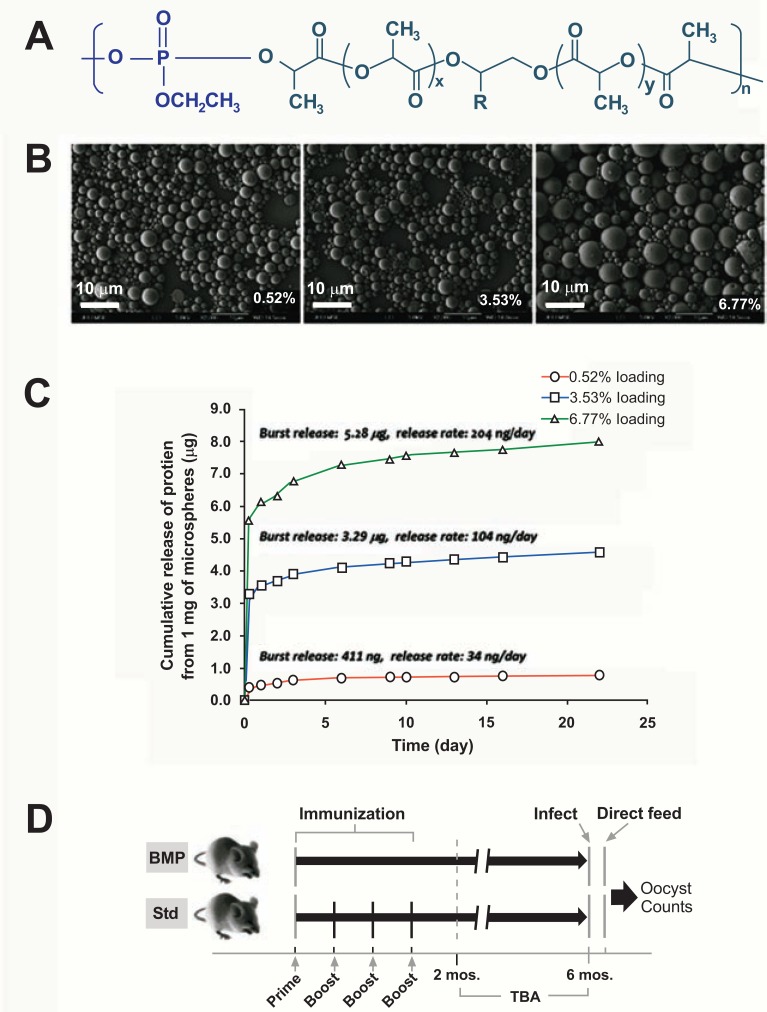
**Polylactofate biodegradable microparticles for single inoculation delivery of a malaria transmission-blocking vaccine antigen.** (**A**) Structure of polylactofate (PLE). (**B**) Scanning electron micrographs of three batches of BMPs with 0.52%, 3.53% and 6.77% protein loading, respectively (Scale bars = 10 µm). (**C**) Effect of protein loading level on the cumulative release profile of encapsulated proteins from BMPs. (**D**) Immunization dosing regimens for BMP and control groups, and functional analysis by direct feeding assay (DFA).

Recombinant APN1 was produced in *E. coli *as previously described [23]. Polylactofate (PLE) was used to prepare BMPs. PLE is a poly(lactide-co-glycolide) derivative with good biocompatibility and better control of biodegradation rate and physical properties [50, 51] (Fig. **1A**). BMPs were prepared by a modified double emulsion method [50], and characterized by scanning electron microscopy. The release kinetics of APN1 from BMPs was characterized by monitoring the concentration of APN1 using ELISA. To modulate APN1 release, we used bovine serum albumin (BSA) as a filler protein.

### Immunizations

BALB/c female mice were immunized with either (A) recombinant APN1 in PBS in suspension with alum, or (B) recombinant APN1 in PBS emulsified with incomplete Freund’s adjuvant (IFA), or (C) BMP-encapsulated recombinant APN1 delivered with alum, or (D) BMP encapsulated APN1 with IFA or (E) empty BMP with alum or (F) empty BMP with IFA. For all treatment groups, mice received 2 µg antigen/mouse/ dose. At day 0, mice received a subcutaneous (s.c.) injection of the appropriate inoculum in a volume of 100 μl per mouse. At 2, 4 and 6 weeks post priming, mice in the Control cohorts (treatments A and B, above) were boosted intraperitoneally (*i.p.*) with the same dose of the inoculum per mouse, whereas the BMP cohorts were boosted only with PBS. At these time points, each mouse was bled to collect sera for anti-APN1 antibody titer determination *via *ELISA (Fig. **1C**).

### ELISA and Cytokine Assay

ELISAs were performed as previously described, using recombinant APN1 as coating antigen [7]. For cytokine assays, the spleen was removed and homogenized at 10% wt/vol in 2% fetal bovine serum/minimal essential medium, and supernatants stored at -80°C until used. Cytokines were measured in tissue homogenates using bead-based multiplex cytokine kits (Bio-Plex, Bio-Rad), according to manufacturer’s instructions. The limits of detection were as follows: interleukin (IL)-1α, 1.32 pg/ml, IL-1β, 1.70 pg/ml; IL-2, 1.98 pg/ml; IL-3, 1.32 pg/ml; IL-4, 2.43 pg/ml; IL-5, 1.69 pg/ml; IL-5, 1.69 pg/ml; IL-6, 1.02 pg/ml; IL-9, 1.36 pg/ml; IL-10, 1.04 pg/ml; IL-12/23 p40, 1.15 pg/ml; IL-12 p70, 1.20 pg/ml; IL-13, 1.57 pg/ml; IL-17a, 1.44 pg/ml; interferon (IFN)-γ, 1.30 pg/ml; eotaxin, 1.70 pg/ml; granulocyte-colony stimulating factor, 1.69 pg/ml; granulocyte-macrophage-colony stimulating factor, 1.58 pg/ml; monocyte chemo-attractant protein, 1.71 pg/ml; macrophage inflammatory protein (MIP)-1α, 1.57 pg/ml; MIP-1β, 1.20 pg/ml; RANTES, 0.95 pg/ml; tumor necrosis factor (TNF)-α, 1.73 pg/ml. Cytokine measurements below the limit of detection as determined by the standard curve for each individual cytokine were assigned a value of the limit of detection/√2 for statistical analysis and plotting. Statistical significance was determined by One-way ANOVA with Bonferroni Post Test, α = 0.05.

### Transmission-Blocking Assays

The Direct Feeding Assays (DFA) were conducted as previously described [7] at 2 months and at 6 months post-priming immunization (Fig. **1D**). Since *Plasmodium *oocyst numbers are generally overdispersed in our system, statistical significance was assessed using the non-parametric Mann Whitney U Test, α = 0.05.

## RESULTS

**Fig. (2):**
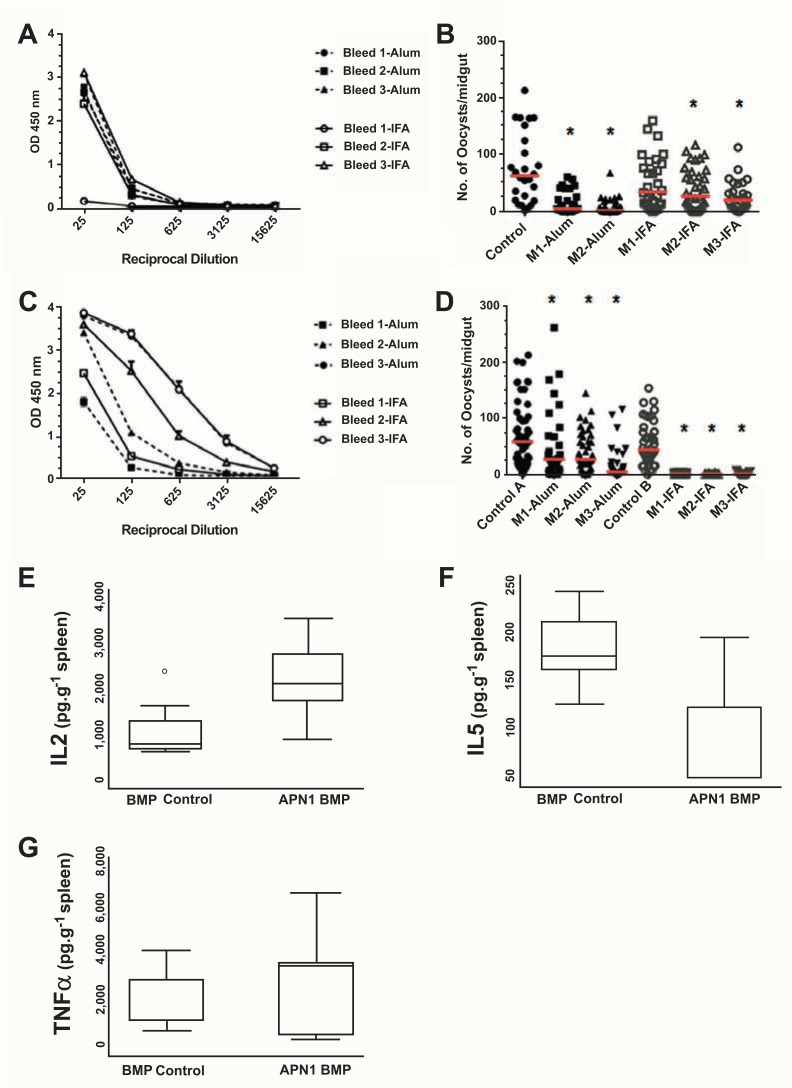
**Characterization of the immune response and activity of antibodies elicited following immunization with APN1.** (**A**) APN1-specific antibody titers (at bleeds 1-3) for mice that received only a single inoculation of BMP encapsulated APN1 with alum or IFA. (**B**) Direct Feeding Assay to assess short-term transmission-blocking potential of mouse APN1 antisera against *Plasmodium berghei* (ANKA 2.34) in *Anopheles gambiae* (KEELE) mosquitoes for groups in (**A**) at two months post-priming immunization (see Fig. **1D**). (**C**) APN1-specific antibody titers (at bleeds 1-3, at two week intervals) for mice that received APN1 with either alum or IFA as adjuvant. (**D**) Direct Feeding Assay to assess short-term transmission-blocking potential of mouse APN1 antisera against *P. berghei* (ANKA 2.34) in *An. gambiae* (Keele) mosquitoes for groups in (**C**) at two months post-priming immunization. For **A-D**: Median oocyst numbers are represented by the horizontal line; control infections were from an agematched, unimmunized mouse; and the P-value was determined by Mann Whitney U Test and asterisks (*) indicate statistical significance at α = 0.05. (**E-G**) APN1-BMP induces pro-T-cell and B-cell cytokines. Twenty-three cytokines measured in homogenized spleen samples from mice that received either BMP (empty) or APN1-encapsulated BMPs. Data expressed on pg/g of tissue basis (corrected for spleen weight). The two significantly different cytokines (**E**) IL-2 and (**F**) IL-5 and one cytokine, TNF-α, which was not significantly different (**G**), are shown. Data presented as box and whiskers plots with outliers identified as dots. Median is the horizontal line within the box. Statistical significance was determined by one way ANOVA with Bonferroni Post Test, α = 0.05.

We generated PLE BMPs (Fig. **1B**) and optimized the protocol for controlling the protein antigen loading levels. We then used loading level as a parameter to adjust the release rate of the antigen. Using bovine serum albumin (BSA) as a model antigen, we have shown that the amount of antigen released from the BMPs can be controlled by loading level as shown in Fig. (**1C**). For example, BMPs with 3.53% protein loading level released protein antigen at a rate of ~ 104 ng/day per mg of BMPs, after an initial burst release of 9.3% of the total protein loaded. These release rates amounted to a release of approximately 15% of total protein within the first 22 days. For this pilot study, we used BMPs with 3.53% of protein loading level. We compared the humoral response of mice using the schedule outlined in Fig. (**1D**). Mice immunized with a single inoculation of APN1-containing BMPs plus IFA or Alum alone (Fig. **2A**) mounted a relatively poor antibody response in comparison to a prime and 3-boost regimen of APN1 plus IFA/Alum (Fig. **2C**). Surprisingly, the immunoglobulin subtypes (IgG1, IgG2a, and IgG2b) generated in the group that received a single immunization of APN1-BMPs/alum were similar to that elicited by the APN1-alum (data not shown).

To determine the short-term and long-term efficacy of transmission-blocking serum antibodies against *P. berghei* we performed direct feeding assays (DFAs) two weeks following the final boost in the control group at 2 months (60 days) and at 6 months (180 days) (Figs. **1D**, **2B**, **D**). We compared parasite development in mosquitoes that were fed on four groups: (i) control cohort (primed with APN1/alum followed by three boosts); (ii) treatment group receiving a single inoculation of APN1-BMPplus alum, (iii) treatment group receiving a single inoculation of APN1-BMP plus IFA, and (iv) control (naïve/unimmunized or empty BMP immunized) infected mice. At 60 days, both the APN1-alum and APN1-IFA immunized controls elicited functional transmission-blocking antibodies against *P. berghei *(Fig. **2B**). Despite the lower antibody titer observed previously, APN1-BMP-immunized mice generated a significant level of functional antibody titers that can effectively inhibit oocyst development in *An. gambiae* (Fig. **2C**, **D**). We observed that at 6 months post-priming immunization, serum from mice immunized with APN1/alum, following a standard immunization regimen, no longer contained any transmission-blocking antibodies [refer to median oocyst number/prevalence for APN1-Alum Control (M3)]. In contrast, individual mice that received either APN1-BMPs/alum or APN1-BMPs/IFA still retained functional transmission-blocking antibody (Table **3**). Cytokine levels analyzed by multiplex assay also demonstrated that APN1-BMPs significantly induced splenic pro-T-cell and B-cell cytokines such as IL-2 and IL-5. These data suggest a cell-specific immune effect rather than a general inflammatory process in response to BMP dosing, thereby validating the specificity of the immune response to the vaccine formulation (Fig. **2D-F**).

## DISCUSSION

Although nano- and microparticle technology has been already shown to potentiate the immune response to pathogen-derived antigens [52, 53], including malaria [44, 45, 49, 54], its use in TBV delivery while previously postulated [9], remained relatively untested [44]. Our small scale study adds to the growing body of data, and moreover, successfully demonstrates that (1) APN1-BMPs with alum adjuvant elicit antigen-specific antibody titers after single dose immunization and induce the production of cell-activation rather than broad-spectrum pro-inflammatory cytokines; (2) the functional transmission-blocking activity of APN1 antisera against *P. berghei* from mice immunized with a single dose of APN1-BMP in *An.* gambiae mosquitoes; and (3) that with a potent adjuvant such as incomplete Freund’s adjuvant, immunization with BMPs elicits and maintains transmission-blocking titers in mice for 6 months. Furthermore, the protracted release kinetics of model antigen over 16 days *in vitro *by our PLE BMP demonstrates a more controlled profile as compared to gel core liposome or conventional liposome particles which have been shown to exhibit a 50% cumulative percentage release of antigen at 10-15 days and 5 days, respectively [44]. These data suggest that larger microparticles allow for enhanced control over the release profile. Recently, it was shown that incorporation of TLR9 agonists in 1-µm gel core liposomes can significantly enhance the immune response to the poorly immunogenic Pfs25 SSM-TBV antigen [44]. Thus, the use of molecular adjuvants as filler molecules may also be considered in future formulations. Taken together with our proof-of-concept data, we anticipate that co-encapsulation of adjuvant and administration of different BMPs with different release profiles (e.g. burst and fast release serve as priming and sustained/delayed release as boosting dose) will significantly enhance the overall immune responses.

## CONCLUSION AND FUTURE PERSPECTIVES

Vaccines are traditionally developed with the prospect of eventual parenteral administration, and the TPP for SSM-TBVs suggests that this is the primary consideration for the development of the leading candidates (Table **1**). Given the uniqueness of the SSM-TBV approach it is argued that non-classical concepts for vaccine delivery may be more suitable. In this section we highlight some concerns surrounding the use of NPs and BMPs when considering vaccine delivery not only through parenteral, oral or mucosal routes, but specifically *via *cutaneous immunization.

### Does Size Matter?

It has been shown that 40 nm polystyrene nanoparticles (NP) that are surface-coated with antigen can be targeted to the lymph nodes to generate a robust immune response [46-48, 55]. NPs have also been shown to increase the breadth and avidity of the humoral response to a *Plasmodium vivax* blood stage antigen [37, 45] arising in part through a synergistic effect of surface displayed and encapsulated antigen in a single formulation. However, it is likely that the nature of the particle, the characteristics of the antigen, including intrinsic immunogenicity and molecular size, presence of conformational antibody epitopes, as well as the type of immune response that should be engendered will have a direct influence on the selection of biodegradable nanoparticles (BNP) *vs* BMP as carrier (reviewed in [56, 57]). It was found that larger particles engender a Type 2 response while smaller, virus-sized particles induced a largely cell-mediated Type 1 response [46]. An interesting approach would be to use different BNP and BMP carriers, leveraging the advantages of antigen targeting and antigen depot effect endowed by each type of particles to reach a specific immune response endpoint [36]. In the context of SSM-TBVs, it remains to be seen if different carrier modes can further potentiate the humoral response to confer long-term protection.

### Does Route of Delivery Matter?

It has been shown that size also has a direct influence on the effectiveness of delivery when the route of administration is considered. Intradermal or subcutaneous inoculation of BNPs and BMPs bypasses the issue of tissue barriers and proteolytic environments, in the case of oral administration. However, the clear potential of this technology lies in the idea of needle-free vaccination. The use of BNP and BMPs as carriers for transcutaneous or cutaneous immunization has been extensively studied [57, 58] and it is well recognized that the main barrier for trans- or percutaneous delivery of antigen payload to the rich population of APCs in the epidermis and dermis is the *stratum corneum* lipid bilayer overlaying the epidermis [57]. Passive diffusion of antigen carried *via *nanocarriers through intercellular or follicular routes to access to the APCs in the epidermis and preferably the dermis has been demonstrated, strongly implying that presentation is size dependent [58].

While there are clear opportunities for the utility of BNPs and BMPs in the development of the next generation of SSM-TBVs, the current working model by many vaccine developers remains generally conservative. This is rightly so, since malaria vaccines must be low cost to allow for general distribution. The huge number of vaccine doses to cover the more than one third of the world’s population is likely to be borne by public-private partnerships and other novel funding models. However, there is hope for this approach since the prevailing strategy has been more recently revisited by the PATH Malaria Vaccine Initiative [59]. One of the biggest benefits of the BNP/BMP approach, namely the potential to mimic natural boosting, is quite attractive, especially in light of the prediction that titers of antibody (produced either naturally or following vaccination) against sexual stage and mosquito antigens will likely wane over time [60]. Furthermore, there is optimism that by leveraging the potential advantages conferred by particle-based approaches, the community will ultimately see the incorporation of vaccine antigens targeting different life stages of the parasite in a single particle formulation.

## Figures and Tables

**Table 1.** The Proposed Target Product Profile (TPP) for a Malaria Sexual Stage and Mosquito Transmission-Blocking Vaccine (SSM-TBV) [61]

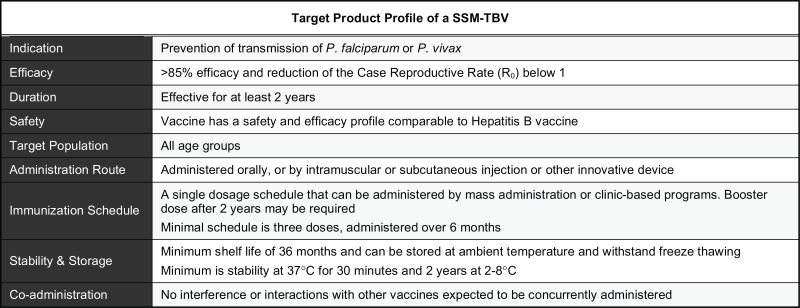

**Table 2.** Update of the Current Status and Characteristics of the Leading SSM-TBV Candidates

Target Antigen	Current Status	Attributes

**P230**	Recombinant antigen expression through a variety of systems including plant, cell free wheat germ systems.	Present in the gametocyte and can confer natural boosting [10, 19]
		Immunogenicity is poor and requires a strong adjuvant [19, 62, 63]
		Molecule is large, resulting in difficulty in expression and maintenance of conformational epitopes [63]

**P48/45**	Recombinant antigen expression using *E. coli* (codon harmonized)	Conformational epitopes necessitates an appropriate expression system [33]
		Immunogenic protein in animals (alum) and is further enhanced by using a strong adjuvant [33]

**P25**	Phase I clinical trials	Immunogenic varies depending on route [63] but is generally considered poorly immunogenic by itself and may require a strong adjuvant [29, 64-66] or conjugation to a molecular adjuvant or protein carrier [67]
	+ Conjugated to recombinant *Pseudomonas aeruginosa *ExoProtein A [62]	Reactogenic formulations prevented continuation of the first Phase I clinical trial [65]
	Phase I Clinical trial of ExoProtein A product is ongoing	Successfully produced the small immunogen in yeast and plants [68]

**APN1**	Entering Process Development	Immunogenic in mice [7] and non-human primates (Dinglasan, unpublished) using alum as adjuvant
		Does not require an adjuvant for complete seroconversion in mice [7]

**Table 3.** Direct Feeding Assays (DFA) to Assess Long-Term Transmission-Blocking Potential of Mouse APN1 Antisera Against *Plasmodium berghei* (ANKA 2.34) in *Anopheles gambiae* (Keele) Mosquitoes. DFAs were Performed at 6 Months Post-Priming Immunization (see Fig. 1D)

Group (Mouse #)	N	Median Oocyst # (Range)	% Inhibition	Prevalence	*P*-Value
*Long-Term*					
APN1-Alum Control (M3)	23	82 (1-181)	—	100%	—
APN1-BMP-Alum (M4)	22	8.5 (0-84)	90	82%	<0.0001
APN1-BMP-Alum (M5)	*n.d.*	—	—	—	—
APN1-BMP-IFA (M4)	32	16 (0-124)	81	59%	<0.0001
APN1-BMP-IFA (M5)	22	0 (0-1)	100	9%	<0.0001

Groups: APN1-Alum Control = recombinant APN1 + alum, using a prime + 3 boost immunization regimen (age-matched with BMP groups); APN1-BMP =APN1-BMP + alum (single inoculation); APN1-BMPIFA=APN1-BMP +Incomplete Freund's adjuvant (single inoculation). *n.d*., not determined since the mouse did not survive the mosquito feeding. P-value determined by Mann Whitney U Test, α = 0.05.

## ABBREVIATIONS

BMP: = Biodegradable microparticle
NP: = Nanoparticle
TBV: = Transmission-blocking vaccine
SSM-TBV: = Sexual stage and mosquito TBV
APN1: = Alanyl aminopeptidase N 1
PLGA: = Poly-lactic-co-glycolic acid
FCA: = Freund’s complete adjuvant
IFA: = Incomplete Freund’s adjuvant.
